# Immunoglobulin M: An Ancient Antiviral Weapon – Rediscovered

**DOI:** 10.3389/fimmu.2020.01943

**Published:** 2020-08-11

**Authors:** Siqi Gong, Ruth M. Ruprecht

**Affiliations:** ^1^New Iberia Research Center, University of Louisiana at Lafayette, New Iberia, LA, United States; ^2^Department of Microbiology, Immunology and Molecular Genetics, The University of Texas Health Science Center at San Antonio, San Antonio, TX, United States

**Keywords:** IgM structure, IgM function, recombinant monoclonal IgM, passive mucosal immunization with IgM, prevention of mucosal virus transmission by IgM, vaccine-induced long-lived IgM plasma cells

## Abstract

Recent discoveries have shed new light onto immunoglobulin M (IgM), an ancient antibody class preserved throughout evolution in all vertebrates. First, IgM – long thought to be a perfect pentamer – was shown to be asymmetric, resembling a quasi-hexamer missing one monomer and containing a gap. Second, this gap allows IgM to serve as carrier of a specific host protein, apoptosis inhibitor of macrophages (AIM), which is released to promote removal of dead-cell debris, cancer cells, or pathogens. Third, recombinant IgM delivered mucosally by passive immunization gave proof-of-concept that this antibody class can prevent mucosal simian-human immunodeficiency virus transmission in non-human primates. Finally, IgM’s role in adaptive immunity goes beyond being only a first defender to respond to pathogen invasion, as long-lived IgM plasma cells have been observed predominantly residing in the spleen. In fact, IgM produced by such cells contained somatic hypermutations and was linked to protection against lethal influenza virus challenge in murine models. Importantly, such long-lived IgM plasma cells had been induced by immunization 1 year before challenge. Together, new data on IgM function raise the possibility that vaccine strategies aimed at preventing virus acquisition could include this ancient weapon.

## Introduction

Immunoglobulin M (IgM) is the first responder to foreign invaders – including viral pathogens that cause major pandemics. It is the only antibody class that exists in all vertebrate animals (1). Its monomeric form is expressed on B cells as the B-cell antigen receptor. When secreted, IgM is predominantly pentameric and contains the joining chain (J chain). In humans, IgM is present at a relatively high concentration in serum (∼1.47 mg/ml) (2). The J chain allows IgM to be transported across mucosal epithelia through binding with the polymeric immunoglobulin receptor (pIgR), an interaction that leads to the formation of secretory IgM (3).

Because IgM is the first antibody response in viral infections, this Ig class has important value for diagnosis. IgM’s pentameric structure prevents passage across the placenta. Consequently, viral infections of the fetus or newborn are recognized by IgM responses against the background of transplacentally transferred maternal IgG.

IgM’s multimeric structure is well suited to bind viral surface proteins. The high avidity may also allow IgM to better tolerate mutations in viral targets – an important consideration for viral pathogens with high mutation rates. IgM is also a potent complement activator. However, despite IgM’s unique characteristics, its role in the prevention and treatment of viral infections remains understudied. The goal of this review is to give an overview of recent data regarding IgM structure, function, and IgM’s role in acute and longer-lasting antiviral host defenses against virus acquisition.

## IgM Structure

**FIGURE 1:**
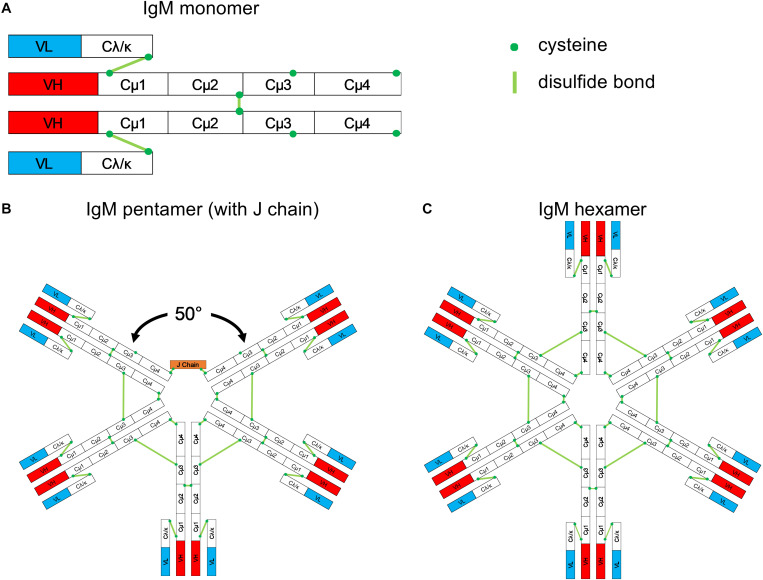
Schematic structure of IgM. **(A)** Monomeric IgM is composed of two heavy (μ) and two light (λ/κ) chains. Each heavy or light chain contains one variable region (VH or VL) and one constant region (Cμ1-4 and Cλ/κ). **(B)** Pentameric IgM contains five monomers and one J chain; disulfide bonds between each monomer form the pentamer; the structure shown in **(B)** is based upon the recent EM image presented by Hiramoto et al. (12). There is a 50° gap where the J chain resides. **(C)** The IgM hexamer contains six monomers and resembles a hexagon. The J chain is generally absent in hexamers.

Monomeric IgM consists of two heavy (μ) and two light (L) chains, like monomers of all other antibody classes. The μ chain constant region contains four domains (Cμ1-Cμ2-Cμ3-Cμ4) and a C-terminal tailpiece (Figure 1A). The Cμ2 domain in the μ chain replaces the hinge region found in the heavy chains of IgG, IgD, and IgA that provides rotational flexibility of the fragment antigen-binding (Fab) domains in these heavy chains (4). However, the lack of a hinge region does not imply that IgM molecules lack flexibility (5). Monomeric IgM is mostly expressed as a surface-bound receptor on B cells, and it is essential for B-cell development. When secreted, IgMs are predominantly polymers in healthy individuals. However, monomeric IgM is frequently secreted in patients with autoimmune diseases (6, 7).

Multiple IgM monomers assemble through interchain disulfide bridge formation between cysteines in the Cμ2, Cμ3, and the tailpiece to form polymeric IgM. In the plasma of humans and mice, the pentameric form is the most abundant IgM version. It contains five monomers and an additional small protein, the joining (J) chain, which bridges the cysteine residues within the tailpiece of two neighboring IgM monomers (8, 9).

The most widely accepted structure of the IgM pentamer is a symmetrical pentagonal structure based upon negative-stain electron microscopy (EM) (10, 11). In 2009, Czajkowsky and Shao (4) proposed that pentameric IgM is a non-planar, mushroom-shaped structure based upon cryo-atomic force microscopy (cryo-AFM); in this model, the C-terminal regions protrude. Most recently, Hiramoto et al. (12) suggested a novel, asymmetric structure based upon single-particle EM images of negatively stained IgM pentamers; in this model, pentameric IgM resembles a quasi-hexamer that is missing one of the monomers – akin to a tooth gap (Figure 1B). Interestingly, when monoclonal IgM was generated by cotransfection of heavy and light chain expression plasmids in the absence of an expression plasmid for the J chain, the most abundantly produced IgM consisted of symmetrical hexamers (Figure 1C) and a lower fraction of symmetrical pentamers; “tooth-gap” IgM structures were notably absent. These findings imply that the incorporation of the J chain results in pentamers with gaps. The asymmetric structure of pentameric IgM was confirmed recently by high-resolution cryo-EM images (13). Taken together, recent structural analyses revealed pentameric IgM to be a non-planar, mushroom-shaped, asymmetric pentamer with protruding C-terminal regions, and a J chain resulting in the formation of gapped structures. Antibody-secreting cells can produce both pentameric and hexameric IgM. The abundance of intracellular J chains is positively correlated with the formation of pentamers; the more J chains are expressed by the cell, the higher the ratio of pentameric to hexameric IgM (9, 14).

Due to its multimeric structure, IgM has a high valency compared to other immunoglobulins. This characteristic allows IgM to compensate for low affinity to a degree and to bind target antigens with high avidity. Multimeric IgM also binds antigens with repeated epitopes efficiently and results in agglutination of bacteria, red blood cells, and viruses (15, 16).

## Pentameric IgM Crosses Mucosal Epithelia

The presence of the J chain allows pentameric IgM to interact with the pIgR expressed on the basolateral surface of epithelial cells. This interaction leads to the formation of an IgM-pIgR complex that is transported across the cell to the luminal side where pIgR is proteolytically cleaved. A pIgR fragment, termed secretory component (SC), remains bound to the pentameric IgM giving rise to secretory IgM. Thus, the pIgR-mediated transport across the epithelial layer grants IgM access to the mucosal lumen (3). The interaction of secretory IgM with commensal bacteria in mice and humans and its role in inducing tolerance have recently been reviewed (17).

## Asymmetric Pentameric IgM Sequesters a Host Protein

The gap caused by the J chain in the structure of pentameric IgM can be filled by a specific host protein, apoptosis inhibitor of macrophage (AIM, encoded by the *cd5l* gene), according to EM images by Hiramoto et al. (12). AIM belongs to the scavenger receptor cysteine-rich (SRCR) superfamily and is mainly produced by tissue macrophages. Due to its relatively small size (37 kD in humans), free AIM can be filtered easily by the glomerulus. Binding to IgM not only protects AIM from renal excretion but also inactivates AIM (18–20). Under various disease conditions, especially acute kidney injury, AIM can be released from its IgM carrier either locally or systematically to inhibit apoptosis of thymocytes and other cell types (21), and to facilitate the removal of excess fat, bacteria, cancer cells, or dead-cell debris (22).

When AIM-containing IgM binds antigen and forms immune complexes (ICs), AIM’s presence affects the interaction of the resulting IgM-ICs with Fcα/μR expressed on the surface of follicular dendritic cells (FDCs). As a result, internalization of the IgM-ICs is slowed, thus prolonging their surface retention. This facilitates antigen presentation to B cells and stimulates secondary antibody responses (18).

## Multimeric IgM Is a Potent Complement Activator

After binding to a surface-exposed antigen, multimeric IgM shifts its conformation to a staple-like arrangement (10). During the conformational change, both IgM pentamers and hexamers adopt hexagonal arrangements, thereby exposing their C1q-binding sites. This leads to complement binding and activation of the complement cascade (13), thereby lysing virions or virus-infected cells, which accelerates cellular and humoral immune responses (23). Sharp et al. (13) found that complexes formed by surface-bound multimeric IgM and complement C1 are more structurally homogenous and orderly than those consisting of surface-bound, hexamerized IgG and C1. When compared to IgG, IgM is far superior as complement activator; a single antigen-bound IgM molecule can activate complement and lyse red blood cells, while a thousand or more IgG molecules are required to achieve the same (24, 25).

## IgM Regulates the Immune System Through Its Interaction With Fc Receptors

Other than pIgR, IgM can interact with two other Fc receptors, Fcα/μR, and FcμR. Fcα/μR, an Fc receptor closely related to pIgR, can interact with both IgA and IgM. Unlike pIgR, which is expressed only on epithelia, Fcα/μR is expressed on both hematopoietic and non-hematopoietic cells in the liver, kidney, small and large intestines, testis, and placenta (26). In mice, it is expressed on macrophages, FDCs, marginal zone (MZ) and follicular B cells, but not on T cells, NK cells, or granulocytes (26, 27). In humans, Fcα/μR is expressed predominantly on germinal center FDCs (28). IgM-coated microorganisms or IgM-antigen ICs bind to Fcα/μR and are phagocytosed by B cells *in vitro*. This process could facilitate the processing and presentation of antigens to T-helper cells for the induction of immune response against T-dependent antigens (27). However, Fcα/μR also negatively regulates complement receptor-mediated antigen retention by MZ B cells and suppresses immune responses against T-independent (TI) antigens. Because various self-antigens are TI antigens, natural IgMs suppress autoimmune responses by forming ICs with these antigens. The resulting ICs are then phagocytosed through interaction with the Fcα/μR (29).

FcμR is an Fc receptor specific for IgM; other names for FcμR include Fc fragment of IgM receptor (FCMR) and Toso/Fas apoptotic inhibitory molecule 3 (FAIM3) (30). It is expressed by B cells, T cells, and NK cells in humans, but only by B cells in mice (31–33). It has a high affinity to the Fc portion of multimeric IgM (∼10 nM), but a lower affinity to monomeric IgM (31). When IgM binds to a membrane component on the same cell surface via its Fab, it binds more efficiently to FcμR (34). The function of FcμR is still under investigation. Studies on FcμR-deficient mice suggested that this receptor broadly regulates cellular activation and controls autoantibody production (32, 35–37).

## Both Natural and Adaptive IgMs Protect Against Viral Infections

There are two types of secreted IgM (sIgM), natural (innate) and adaptive IgM; the latter is also termed “immune” or “induced” IgM. Even without apparent antigenic stimulation, circulating antibodies are present in human cord blood (38) and in the blood of germ/antigen-free mice (39, 40). Such spontaneously produced antibodies are called natural antibodies (41), and the IgM class predominates (42) although IgG and IgA natural antibodies have been described (43, 44). The repertoire of natural antibodies is highly conserved (38, 40, 45, 46). In mice, natural IgM is mostly produced by the long-lived, self-renewing CD5^+^ B1 subset of B cells (47) in the spleen, bone marrow, and peritoneal cavity (48–50). B1 cells generally harbor germline versions of the V gene segments with limited somatic hypermutation (51–53). Natural IgMs often display low affinity and polyreactivity. However, natural IgM – despite its low affinity – plays a significant role in the primary host defense.

Natural IgM is broadly reactive to not only protein but also non-protein antigens, such as phosphorylcholine (54), phosphatidylcholine (55), and glycans (56, 57). Due to its polyreactivity, natural IgM can recognize foreign antigens without ever having encountered them, making it the very first line of defense against invaders. Indeed, sera from naïve mice contained IgMs able to bind to vesicular stomatitis virus (VSV), lymphocytic choriomeningitis virus (LCMV), and vaccinia virus (58). The anti-VSV IgM antibodies were neutralizing and strain-specific as shown by cross-absorption with another strain of VSV. In mice lacking sIgM but able to produce all other Ig classes (sIgM^–/–^), viruses disseminated much faster than in wild-type (WT) mice during early-stage infection. Natural IgM trapped virions in the spleen and reduced blood-borne viral trafficking to non-lymphatic organs, such as the kidney. When serum from naïve WT mice that contained predominantly natural IgM was passively transferred to sIgM^–/–^ mice, the recipients were protected against lethal VSV challenge. Moreover, Ochsenbein et al. (58) suggested that natural IgM influences not only early virus dissemination but also delivers trapped virus particles to secondary lymphatic organs, thereby accelerating and enhancing adaptive immune responses – based upon the known, delayed neutralizing IgG responses in sIgM^–/–^ mice (58).

Natural IgM in the sera from naïve mice can also neutralize and aggregate influenza virus in a complement-dependent manner. The infusion of this natural IgM to RAG1^–/–^ mice, which lack both mature B cells and T cells, provided a modest degree of protection against influenza virus challenge (59).

Naturally occurring anti-leukocyte IgMs isolated from both normal and HIV-infected individuals that bind to CD4, CCR5, and CXCR4 also have been reported by Lobo et al. to inhibit HIV-1 infection in cultured human primary cells and cell lines (60). The same anti-leukocyte IgMs were also tested for their anti-HIV activity in a humanized severe combined immunodeficiency (SCID) mouse model. These animals, reconstituted with human peripheral blood lymphocytes (PBLs), were protected by intraperitoneal (i.p.) injection of these natural IgMs from i.p. HIV challenge (60) [review in (61)]. These data by Lobo et al. imply that anti-cellular IgM that targets HIV receptors/coreceptors as opposed to directly binding to viral proteins may have beneficial effects.

In summary, natural IgM uses the following three mechanisms to defend against viral infections: (i) neutralization by direct binding to viral proteins or viral receptors expressed on potential viral target cells, with or without complement; (ii) trapping of virions by aggregation; and (iii) transporting viral antigens as ICs to lymphoid tissues, thus promoting adaptive immune responses. The latter most likely occur through complement activation or Fc receptor interaction.

In contrast to natural IgM, adaptive IgM is the first antibody class produced by the body in response to an invading pathogen and is mostly produced by B2 cells in the spleen and lymph nodes. Adaptive and natural IgMs are structurally and functionally similar despite the different producer B cells; the only difference is that natural IgM possesses more flexible antigen-binding sites that provide broader reactivity to various antigens (62–64). Although adaptive IgM is generally not thought to play a significant role in long-term humoral immunity, the recent discovery of long-lived IgM plasma cells (65, 66) suggests that adaptive IgM may be an overlooked contributor in humoral immune defenses against viral infections. Somatic hypermutations have been found in cells producing adaptive IgM (65).

The role of natural and/or adaptive IgM against virus challenges was examined by several groups. Interestingly, IgM produced by both B1 and B2 cells was required to provide full protection against influenza virus challenge in mice (67); natural IgM was only partially protective in this study. Diamond et al. (68) examined the role of IgM against West Nile virus (WNV) in mice, especially viral dissemination into the central nervous system (CNS). WNV mortality rates were compared between WT and sIgM^–/–^ mice that, as mentioned above, do not produce sIgM but can secrete other immunoglobulin classes/isotypes. All of the WT mice survived, in contrast to only 25% of the sIgM^–/–^ mice (68). When WT mice were exposed to WNV for 4 days, neutralizing IgM (adaptive IgM) but no WNV-specific IgG was detected. Serum was collected from these mice with early-stage WNV infection, heat-inactivated, and passively transferred to sIgM^–/–^ mice. Nine out of ten of the latter survived subsequent lethal WNV challenge. In contrast, passive administration of sera from naïve mice did not provide any protection, suggesting that adaptive IgM, but not natural IgM was responsible for the protection against WNV infection. Protective adaptive IgM responses have also been found against polyomavirus (69), VSV (70), rabies virus (71), and influenza virus (72–74).

Recently, Shen et al. (74) isolated a neutralizing monoclonal antibody against influenza virus, termed C7G6, from immunized mice by hybridoma technology. The authors then constructed IgM and IgG1 versions of C7G6. Interestingly, the C7G6-IgM provided more potent and broader neutralization against influenza B strains compared to the IgG1 counterpart *in vitro* and better protection against different strains of influenza virus in mice and ferrets (74). Together, these findings suggest that adaptive IgM might be able to play a significant role in the immune defense against viral infections in general.

## Adaptive IgM Contributes to Long-Term Protection Against Pathogens

According to conventional views, adaptive IgM is produced only during acute infection, is short-lived, and is not associated with hypermutation/affinity maturation. However, Racine et al. (66, 75) demonstrated that infection with the intracellular bacterial pathogen, *Ehrlichia muris*, induced a robust, antigen-specific IgM plasmablast response. These IgM-secreting cells were detectable as late as 1 year after infection, were long-lived, and resided in spleen and bone marrow. Furthermore, all mice deficient of activation-induced cytidine deaminase (AID), which can only produce IgM but none of the other Ig classes due to impaired ability to undergo class-switching and somatic hypermutation, survived *E. muris* challenge. In contrast, 80% of mice lacking all secreted Ig classes succumbed to the challenge, suggesting that IgM alone can protect against *E. muris* infection. To determine the duration of IgM-mediated protection, the authors exposed AID-deficient mice to a different, more pathogenic bacterial strain, *Ixodes ovatus ehrlichia* (IOE). The re-exposure occurred 250 days after the initial *E. muris* infection, from which all mice had recovered; the majority of them survived IOE rechallenge, while none of the naïve mice did. These data suggest the *E. muris*-induced, IgM-mediated immunity was long-lasting. To exclude the possibility that the long-lasting IgM responses required bacterial persistence, the authors treated the *E. muris* infection with an antibiotic. After this, titers of *E. muris-*specific serum IgM were significantly reduced, whereas the IgM-secreting cell population persisted at only slightly lower numbers. These IgM-secreting cells still protected 75% of the mice against lethal IOE challenge 77 days after the initial *E. muris* infection. In contrast, none of the naïve mice survived, regardless of prior antibiotic treatment. In this mouse model, *E. muris* infection functioned akin to a live attenuated vaccine that induced antigen-specific IgM responses that cross-protected against lethal IOE rechallenge. Of note, these *E. muris-*induced IgM responses persisted for months and did not require the chronic presence of *E. muris*.

More recently, Bohannon et al. (65) showed that immunizing mice with inactivated influenza virus induced long-lived, antigen-specific IgM-secreting plasma cells that persisted in the spleen for 2 years. The same type of cells was also observed in mice exposed to live influenza virus or LCMV. These long-lived IgM plasma cells could develop independently of germinal centers (GCs) and were somatically mutated in an AID-dependent manner. Interestingly, after adoptive transfer to naïve recipient mice, these plasma cells were relatively long-lived and had an estimated half-life of 86 days, which did not differ significantly from that of IgG plasma cells in this study (*t*_1/2_ = 145 days; *p* = 0.82) (65).

Next, Bohannon et al. (65) immunized WT C57/BL/6 mice once with inactivated influenza virus and treated them with a cytotoxic antibody targeting the CD40 ligand (CD40L) on days 6, 8, and 10 after vaccination to deplete T-helper cells. The anti-CD40L antibody treatment was designed to disrupt GC formation, to prevent Ig class switching, and thus to block the development of long-lived IgG plasma cells (76). Indeed, the influenza virus-specific, long-lived IgG plasma cells were ablated (65), and antiviral neutralizing antibodies in sera taken 150 days post-immunization were predominantly IgM. One year after immunization, these immunized, anti-CD40L antibody-treated mice were challenged intranasally with a lethal dose of influenza virus. While all unimmunized controls succumbed to the infection, 80% of immunized and antibody-treated mice survived, thus suggesting that the long-lived IgM plasma cells and adaptive IgM provided sufficient protection in the absence of long-lived IgG plasma cells and IgG antibodies. Of note, the authors did not measure IgA-related antiviral responses; thus, the contribution of the latter in the protection against influenza was not excluded, although the anti-CD40L antibody treatment likely also prevented class switching to IgA. It also would be interesting to see whether adoptively transferring virus-specific, long-lived IgM plasma cells to naïve mice would protect against influenza virus challenge. Such an adoptive transfer would eliminate confounding variables, thereby providing proof that IgM responses alone suffice.

Taken together, the data summarized in this section suggest that adaptive IgM contributes to the humoral memory and long-term protection against pathogen infections. The findings also imply that vaccination could potentially induce long-term protective IgM responses.

## Adaptive IgM Responses During Human Immunodeficiency Virus (HIV) Infection

Adaptive IgM is the first virus-specific antibody class to emerge after HIV infection – like in any infection. In 1987, Cooper et al. (77) reported that serum anti-HIV IgM was detectable within an average of 5 days after the onset of acute illness, peaked at 24 days, and became undetectable at 81 days. IgG antibodies first appeared later at 11 days, peaked at 133 days, and remained measurable in all subjects (77).

A more detailed analysis of the time course of initial antibody responses to acute HIV infection was conducted by Tomaras et al. (78); the first detectable antibodies usually were present as IgM-virion ICs, appearing as early as 5 days (median, 8 days) after the day plasma viral RNA became measurable (*T*_0_), which was approximately 10 days after virus transmission. The first free plasma anti-HIV antibodies were anti-gp41 IgM detected in 41% of subjects at a median of 13 days (range, 5 to 18 days) after *T*_0_. These responses decreased over 20 to 40 days, while IgG responses rose over the same period (78).

Of note, the inclusion of anti-HIV IgM detection in the third-generation test kits (IgG/IgM sensitive) shortened the window period, i.e., the time before HIV infection can be diagnosed serologically. Testing for HIV-specific IgM could give positive readouts at a median of 23 days after infection compared to 31 days by tests measuring only IgG (79).

## Recombinant Anti-HIV IgM Protects Mucosal Simian-Human Immunodeficiency Virus (SHIV) Transmission

Unlike anti-HIV IgG or IgA, anti-HIV IgM has remained relatively understudied other than developing it as a diagnostic tool. Recently, we sought to investigate the potential of anti-HIV envelope IgM in preventing virus acquisition in a non-human primate model. We chose passive immunization as our tool because it restricts protection solely to the passively administered antibody and thus establishes a cause-and-effect relationship between the antibody and protection. We first constructed a recombinant human monoclonal anti-HIV IgM using the variable genes of the high-affinity neutralizing antibody, 33C6-IgG1, which targets the conserved V3 loop crown of HIV gp120 (80). The resulting 33C6-IgM contained the J chain and recognized the same epitope as the original IgG1; essentially, we created the opportunity to directly compare isogenic IgM and IgG1 mAb versions. By surface plasmon resonance, 33C6-IgM bound to HIV gp120 with a faster on-rate and a slower off-rate, resulting in a 52-folder higher affinity compared to 33C6-IgG1. *In vitro*, 33C6-IgM neutralized and captured challenge virus particles significantly better than its IgG1 counterpart.

Next, we assessed the potential of the isogenic 33C6 IgM/IgG1 mAb pair to prevent acquisition of a chimeric simian-immunodeficiency virus (SHIV) that carries HIV-1 *env*. After intrarectal (i.r.) passive immunization, 33C6-IgM prevented viremia in four out of six rhesus monkeys challenged i.r. with a single high-dose of SHIV, while all control animals were highly viremic. The degree of protection was similar after i.r. passive immunization with the IgG1 version of the same mAb. We propose that efficient trapping of infectious virions in the mucosal lumen – i.e., immune exclusion – combined with direct virus neutralization, represent the mechanisms of protection by IgM (81). To our knowledge, this is the first demonstration that a recombinant antiviral IgM can prevent mucosal virus transmission in a relevant animal model. Our data also provide the first proof-of-concept that the IgM class of anti-HIV envelope antibodies is protective *in vivo*.

Subsequently, Devito et al. (82) isolated natural IgM-producing B cells from HIV-negative donors; B-cell clones that recognized the HIV gp120 V3 region were selected and immortalized to produce natural IgM mAbs. In a Transwell plate-based assay, some of these natural IgM mAbs inhibited transcytosis of infectious HIV – suggesting that natural IgM may be able to stop HIV from crossing epithelial cell membranes. These *in vitro* data lend support to the protective mechanisms we proposed in our passive immunization study in the rhesus macaque/SHIV model.

## Adaptive IgM Responses During Severe Acute Respiratory Syndrome Coronavirus 2 (SARS-CoV-2) Infection and Coronavirus-19 Disease (COVID-19)

SARS-CoV-2, the cause of the newest pandemic, continues to spread exponentially in many countries. More than 16 million people have been infected worldwide (83), and many have succumbed to COVID-19. Guo et al. (84) reported that IgM, IgA, and IgG antibodies against SARS-CoV-2 appeared as early as 1 day after symptom onset in some individuals. Both IgM ad IgA antibodies were detectable at a median of 5 days post symptom onset, while IgG antibodies were detectable at a median of 14 days. However, Long et al. (85) reported that both antiviral IgM and IgG were detectable at a median of 13 days following the onset of symptoms. IgM and IgG became detectable at the same time in some patients, whereas in others either IgM or IgG were detectable first. Both antiviral IgM and IgG titers plateaued within 6 days after seroconversion. Serological tests could help diagnose suspected COVID-19 cases with negative PCR results. Inclusion of antiviral IgM responses could increase assay sensitivity. Currently, intense research efforts are focused on defining protective anti-SARS-CoV-2 antibody responses.

## Conclusion

The IgM field is undergoing a major but quiet revolution. There is more to this ancient antibody class than what textbooks have described. To begin with – the structure of sIgM in mice and humans, long thought to be a perfect pentamer – actually has been described as an asymmetric molecule that resembles a quasi-hexamer missing one monomer, thereby creating a “tooth gap.” This new structural feature allows gapped pentameric IgM to serve as host protein carrier. To date, this interaction seems to be specific and restricted to host protein AIM, which is released as needed to promote the removal of dead-cell debris, cancer cells, or pathogens.

Second, IgM has unique features against viral infections, such as high avidity. Some viruses tend to exhibit high mutation rates, leading to the generation of quasispecies and neutralization escape mutants. Neutralizing IgG antibodies may lose their affinity to viral targets as a consequence. However, IgM’s high avidity could compensate for the loss of affinity caused by imperfect matching to mutated target epitopes, as shown for influenza virus (74). As such, antiviral IgMs are expected to neutralize a broader range of viral strains compared to their IgG counterparts.

Finally, there is more to IgM’s role in adaptive immunity than being a first responder only. According to commonly held views, IgM has been thought to participate solely in the initial, acute response to viral infections without playing any role in long-term adaptive immunity. However, recent findings in mice demonstrated that antigen-specific, long-lived IgM plasma cells do exist – preferentially in the spleen as shown by adoptive transfer from immunized mice. The half-life of such antigen-specific IgM plasma cells was not much shorter than that of long-lived IgG plasma cells. Most interestingly, long-lived IgM plasma cells contained somatic hypermutations ascribed to AID action. These cells were linked to protection against lethal influenza virus challenge 1 year after immunization in mice, in the absence of GCs and antigen-specific IgG plasma cells.

Of note, recent data summarized in this review imply that induction of protective, long-term IgM responses may be possible by active immunization. Vaccines that include long-term antiviral IgM responses may possess advantages over traditional IgG responses against fast-mutating viruses, such as HIV and other RNA viruses known to replicate via error-prone viral RNA-dependent polymerases or to frequently recombine with different viral strains. However, generating such long-term antiviral IgM responses through vaccination needs further study. For use in passive immunization against viral pathogens, IgM may have great potential.

## Author Contributions

Both authors listed have contributed to the writing of this manuscript and approved it for publication.

## Conflict of Interest

The authors declare that the research was conducted in the absence of any commercial or financial relationships that could be construed as a potential conflict of interest.
